# Full-Length Coding Sequences for 12 Bovine Viral Diarrhea Virus Isolates from Persistently Infected Cattle in a Feedyard in Kansas

**DOI:** 10.1128/genomeA.00487-15

**Published:** 2015-05-21

**Authors:** Aspen M. Workman, Gregory P. Harhay, Michael P. Heaton, Dale M. Grotelueschen, David Sjeklocha, Timothy P. L. Smith

**Affiliations:** a USDA, ARS, U.S. Meat Animal Research Center, Clay Center, Nebraska, USA; b University of Nebraska Great Plains Veterinary Educational Center, Clay Center, Nebraska, USA; c Cattle Empire, LLC, Satanta, Kansas, USA

## Abstract

We report here the full-length coding sequences of 12 bovine viral diarrhea virus (BVDV) isolates from persistently infected cattle in a feedyard in southwest KS. These 12 genomes represent the three major subtypes of BVDV (BVDV-1a, 1b, and 2a) currently circulating in the United States.

## GENOME ANNOUNCEMENT

Bovine viral diarrhea virus (BVDV) is an important pathogen of cattle that causes respiratory disease, enteritis, and immune dysfunction (1). BVDV is a positive-sense RNA virus of the genus *Pestivirus* in the family *Flaviviridae* (2). The genome is approximately 12.3 kb and consists of a single open reading frame flanked by 5′ and 3′ untranslated regions (UTRs). Based on sequence variation, BVDV isolates have been divided into two genotypes, BVDV-1 and BVDV-2 (3, 4), and several subgenotypes (5, 6), with BVDV-1a, 1b, and 2a currently circulating in the United States (7). BVDV infections in cattle can be acute (transient) or persistent. Persistent infection (PI) develops when a fetus is infected early in gestation, prior to the onset of immunologic competence (8). As a result, PI calves maintain lifelong viremia and continuously shed virus into the environment. Here, we report the full-length coding sequences of 12 BVDV isolates from PI cattle in a feedyard in Kansas.

BVDV strains from 119 PI calves were genotyped with 283-bp Sanger reads of the 5′ UTR. BVDV representatives of the 3 subtypes circulating in the United States were selected for complete genome sequencing. Total RNA was extracted from the plasma of 12 infected cattle using TRIzol LS (Life Technologies) and used as input material for the Illumina TruSeq RNA sample preparation kit (catalog no. RS-122-2001). The samples were processed as specified by the manufacturer’s protocol, with one important modification: BVDV genomes lack a 3′ poly(A) tail, and thus the initial step of poly(A) selection was omitted. RNA was chemically fragmented and then reverse transcribed using SuperScript II and random primers. The resulting cDNA was converted to double-stranded cDNA and subjected to end repair and 3′ adenylation. Index adapters were then ligated to the ends of the double-stranded cDNA. The libraries were PCR amplified, quantified, pooled, and then sequenced on an Illumina MiSeq instrument (Illumina, San Diego, CA), with a 600-cycle kit (version 3) to generate 2 × 300-bp paired-end reads.

Raw sequence reads were trimmed using Cutadapt (9) to remove the indexing adapters. The trimmed reads were imported into the Geneious software (version 8.0.5 [10]) and screened against the UniVec_Core database (NCBI) to remove vector sequences. Assembly of the viral genomes was then accomplished using template-assisted assembly, in which trimmed and filtered reads were mapped to reference BVDV genomes (Singer [accession no. L32875], NY-93 [accession no. AF502399], or Osloss [accession no. M96687]) using the Geneious software. Reads that mapped to the reference genome were then *de novo* assembled and annotated for each plasma sample. The 12 genomes each assembled into a single contig. The genome statistics are shown in Table 1.

**TABLE 1:** *De novo* genome assemblies of 12 BVDV strains from PI cattle

Animal ID^a^	Strain ID	BVDV genotype^b^	Genome size (nt)	5′ UTR (nt)	CDS (nt)	3′ UTR (nt)	GenBank accession no.
AL 6	USMARC-51998	1b	12,227	373	11,697	157	KP941581
MO 113	USMARC-55926	1b	12,212	367	11,697	148	KP941592
MO 125	USMARC-53874	1b	12,224	371	11,694	159	KP941583
MO 18	USMARC-55478	1b	12,192	372	11,697	123	KP941587
MO 80	USMARC-55925	1b	12,224	371	11,697	156	KP941591
OK 104	USMARC-55924	1b	12,224	377	11,697	150	KP941590
TX 55	USMARC-55923	1b	12,250	384	11,697	169	KP941589
UNK 21	USMARC-55922	1b	12,243	372	11,697	174	KP941588
OK 88	USMARC-55477	1a	12,165	370	11,697	98	KP941586
OK 77	USMARC-53875	1a	12,294	372	11,697	225	KP941584
MO 76	USMARC-53873	2a	12,273	373	11,694	206	KP941582
OK 92	USMARC-55476	2a	12,267	371	11,694	202	KP941585

a ID, identification.

b Genotyping based on phylogenetic analysis of the complete coding sequences (CDS) and 5′ UTR.

The complete sequences were compared to one another and those of published BVDV sequences. The nucleotide sequence identities ranged from 69% to 94%, and the amino acid identities ranged from 75% to 97%. With the exception of a 3-nucleotide deletion in the E2 peptide of USMARC-53874, no deletions, insertions, or gene duplications in the coding sequences were detected.

### Nucleotide sequence accession numbers. 

The sequences are deposited in GenBank/DDBJ/ENA under the accession numbers listed in Table 1.
